# Selecting Biological Meaningful Environmental Dimensions of Low Discrepancy among Ranges to Predict Potential Distribution of Bean Plataspid Invasion

**DOI:** 10.1371/journal.pone.0046247

**Published:** 2012-09-25

**Authors:** Gengping Zhu, Matthew J. Petersen, Wenjun Bu

**Affiliations:** 1 College of Environmental Science and Engineering, Nankai University, Tianjin, China; 2 College of Life Sciences, Nankai University, Tianjin, China; 3 Department of Entomology, Cornell University, Geneva, New York, United States of America; University of Western Ontario, Canada

## Abstract

**Background:**

The Bean plataspid (*Megacopta cribraria*) (Hemiptera: Pentatomidae), native to Asia, is becoming an invasive species in North America; its potential spread to soybean producing areas in the US is of great concern. Ecological niche modelling (ENM) has been used increasingly in predicting invasive species' potential distribution; however, poor niche model transferability was sometimes reported, leading to the artifactual conclusion of niche differentiation during species' invasion.

**Methodology/Principals:**

We aim to improve the geographical transferability of ENM via environmental variable selection to predict the potential distribution of Bean plataspid invasion. Sixteen environmental dimensions between native and introduced Bean plataspid populations were compared, and classified into two datasets with different degrees of discrepancy by the interquartile range (IQR) overlap in boxplot. Niche models based on these two datasets were compared in native model prediction and invading model projection. Classical niche model approaches (i.e., model calibrated on native range and transferred outside) were used to anticipate the potential distribution of Bean plataspid invasion.

**Conclusions/Significance:**

Niche models based on the two datasets showed little difference in native model predictions; however, when projecting onto the introduced area, models based on the environmental datasets showing low discrepancy among ranges recovered good model transferability in predicting the newly established population of Bean plataspid in the US. Recommendations were made for selecting biological meaningful environmental dimensions of low discrepancy among ranges to improve niche model transferability among these geographically separated areas. Outside of its native range, areas with invasion potential include the southeastern US in North America, southwestern Europe, southeastern South America, southern Africa, and the eastern coastal Australia.

## Introduction

Ecological niche modelling (ENM) has been used increasingly in predicting invasive species' potential distribution [1]–[4], and other aspects of ecology and evolution [5], [6]. Based on integrating known occurrence with environmental variables, ENM seeks to characterize environmental conditions that are suitable for the species, and then to identify where suitable environments are distributed spatially [7]. The assumptions under which ENMs work are the equilibrium between species' distributions and ecological requirements, dispersal without limitation and niche conservatism [8]. The ecological niche of a species here can be defined as the set of environmental conditions under which it is able to maintain populations without immigrational subsidy [9], [10].

The classical ENM approaches for invasive species prediction are to calibrate niche models in species' native range and then transfer models to identify areas of potential invasion. Niche model transferability here refers to the capability of species' occurrence prediction from niche models projected onto novel areas. Several studies reported poor niche model transferability in predicting species' invasion (e.g., [11]–[14], leading to the conclusion of niche differentiation during species' invasion, which violates one of the key assumptions of ENM (i.e., niche conservatism). However, other studies have demonstrated that such differences might be caused by sampling records [15], [16], environmental datasets [4], [17], [18], or the method of ENM analysis [19], [20]. Since the conclusions of niche differentiation can be due, in part, to poor niche model transferability, we hypothesized that if environmental dimensions were selected that reduced discrepancy between native and introduced populations, then niche model transferability might be improved among these areas. Some studies suggested niche model transferability could be improved by selecting fewer environmental dimensions for niche model calibration [4], [17], [21], [22]. However, fewer environmental dimensions also means fewer constraint on niche characterization (i.e., ENM calibration), which might result in higher commission error (predicted presence in areas of actual absence) (Personal communication with Dr. Peterson, Kansas University), although it indeed shows lower omission error (predicted absence in areas of actual presence).

Many true bugs (Hemiptera: Heteroptera) have extended their distributions remarkably in the last century, and some of them have increased their pest status after introduction beyond the native range [4], [23]–[26]. Native to Asia, the Bean plataspid *Megacopta cribraria* (F.), also known as lablab bug or globular stink bug, is the first species of the family Plataspidae to be introduced into the Western Hemisphere [24]. The establishment was confirmed by specimens collected in northeastern Georgia, and by November 2010, it had been reported in Georgia, North Carolina, South Carolina, Tennessee, and Alabama [26]. The newly established US population has attracted much attention due to the damage caused by the species in its native area. In Asia, Bean plataspid is a serious pest of soybeans [27] where it damages young leaves, stems, and newly developed pods [26]. In China, the pest has caused soybean crop losses of 30–50% percent [28]. More importantly, recently it was found infesting soybean production areas in Georgia and South Carolina [29]. The only recent detections may indicate that Bean plataspid is still in early stages of invasion following introduction. Although the scope of the bugs' status as a US crop pest has not yet been determined, their potential for spread to large soybean producing areas in the US is of great concern [29].

In this study, we aim to improve niche model transferability by selecting biological meaningful environmental dimensions with low discrepancy among ranges (i.e., native and introduced) to predict the potential geographic distribution of Bean plataspid invasion. The environmental dimensions occupied by native and introduced populations were firstly compared, and classified into high and low discrepancy datasets by the interquartile range (IQR) overlap in boxplot. Principal component analysis was used to further compare the classified datasets in reduced dimensions. Classical niche model approaches (i.e., model based on native range and transferring to introduced areas) were then used to compare niche model transferability based on these two environmental datasets. The preferred models were chosen to anticipate the potential distribution of Bean plataspid across North America and the world. Model transferability responding to niche space comparison, and implications for variable selection to improve the niche model transferability were discussed in this study.

## Materials and Methods

### Occurrence data

Species occurrence localities were assembled from the literatures and from specimens deposited in the Institute of Entomology at Nankai University. Localities with ambiguous or unclear descriptions were excluded. All geographically recoverable localities were georeferenced in Google Maps, Gazetteer of China [30] or BioGeomancer (http://bg.berkeley.edu/latest/). Invasive records in the U.S. were largely county-level [31] and converted to points by digitizing the centroid of infested counties in Arc GIS 10 (ArcGIS, Environmental Systems Research Institute, Redlands, CA, USA). This method has been used in previous studies, with models based on random points not differing qualitatively from models based on such centre points [4], [12], [14]. A total of 166 and 132 occurrence records were prepared for native and introduced populations respectively.

Montandon described a species closely related to *M. cribraria*, i.e., *Megacopta punctatissima* (as *Coptosoma punctatissimum*, [32]), before later finding specimens that were intermediate between *M. cribraria* and *M. punctatissima* [33]. In a revision of the family Plataspidae from China, Yang considered *M. punctatissima* to be conspecific with *M. cribraria* [34]. Hosokawa et al. reported that the two species were capable of interbreeding and their offspring were found to reproduce successfully [35]. Herein, we treated *M. punctatissima* to be a junior synonym of *M. cribraria* and considered *M. cribraria* as the taxonomic entity and utilized occurrence records accordingly.

The native 166 occurrence points varied in spatial density due to variable sampling intensity over geography. These data might inflate measures of accuracy for presence-only niche models (e.g., Maxent or GARP, see below) [36]. As a result, and to avoid overemphasizing heavily on sampled areas, we selected points for model calibration using a subsampling regime to reduce sampling bias and spatial autocorrelation. Following Nuñez and Medley [37], we generated models using all available occurrence points and measured spatial autocorrelation among model pseudo-residuals (1 – probability of occurrence generated by model) by calculating Moran's *I* at multiple distance classes using SAM v4.0 [38]. Significance was determined using permutation tests. A minimum distance of 150 km was detected, we therefore created a grid with cell dimensions of 1.5×1.5° and selected the occurrence point that close to the centroid of each grid cell. This procedure reduced the number of native occurrences to 89 points used for model calibration, leaving the remaining points used for model testing. The procedure greatly reduced sampling bias and spatial autocorrelation, resulting in evenly distributed occurrence points across space [37].

### Environmental variables

Environmental variables summarizing aspects of climate, topography and habitat were prepared to represent ecologically important factors known to impact the biological prosperity of Bean plataspid [39]–[41] (Table 1). Climate variables represented by bioclimatic factors of temperature and precipitation were derived from WorldClim [42], and of sunshine from CliMond [43]. Highly correlative variables were not included in the analysis. Topography variable represented by elevation was also obtained from the WorldClim database. Habitat variables were represented by the Normalized difference vegetation index (NDVI) derived from http://edit.csic.es/, re-calculated as the average of values for 12 months. All dimensions were set at a spatial resolution of 2.5 arc-min for analysis.

**Table 1:** Principal components analysis (PCA) of environmental variables associated with occurrence of Bean plataspid.

Dataset *I*		Factor Loading		
Variable	Description	PC-1	PC-2	PC-3
**BIO2**	Mean diurnal temperature range	−0.70	0.54	0.12
**BIO6**	Minimum temperature of coldest month	0.79	0.01	0.57
**BIO7**	Temperature annual range	**−0.83**	0.14	−0.49
**BIO13**	Precipitation of wettest month	0.79	0.03	−0.15
**BIO14**	Precipitation of driest month	−0.77	−0.18	0.46
**BIO15**	Precipitation seasonality	**0.87**	0.24	−0.40
**BIO21**	Highest weekly radiation	−0.23	**0.90**	0.16
**BIO22**	Lowest weekly radiation	**0.87**	0.37	0.09
**Eigenvalue**		4.58	1.34	1.00
**Percentage variance**		57.31	16.80	12.48
**Cumulative percentage variance**		57.31	74.11	86.59

Dataset *II*		Factor Loading		
Variable	Description	PC-1	PC-2	PC-3
**BIO1**	Annual mean temperature	**0.91**	−0.12	−0.29
**BIO3**	Isothermality	0.75	0.45	0.42
**BIO4**	Temperature seasonality	**−0.84**	−0.45	0.22
**BIO5**	Maximum temperature of warmest month	0.58	−0.72	0.13
**BIO12**	Annual precipitation	0.10	0.39	−0.78
**BIO20**	Annual mean radiation	0.66	−0.10	0.26
**DEM**	Elevation	−0.16	**0.82**	0.03
**NDVI**	Normalized difference vegetation index	−0.06	0.54	0.50
**Eigenvalue**		2.88	2.04	1.24
**Percentage variance**		36.02	25.54	15.47
**Cumulative percentage variance**		36.02	61.56	77.04

Eigenvalues for the most important variables (>0.8) are in bold.

### Direct comparison and Principal Component Analysis (PCA)

**Figure 1:**
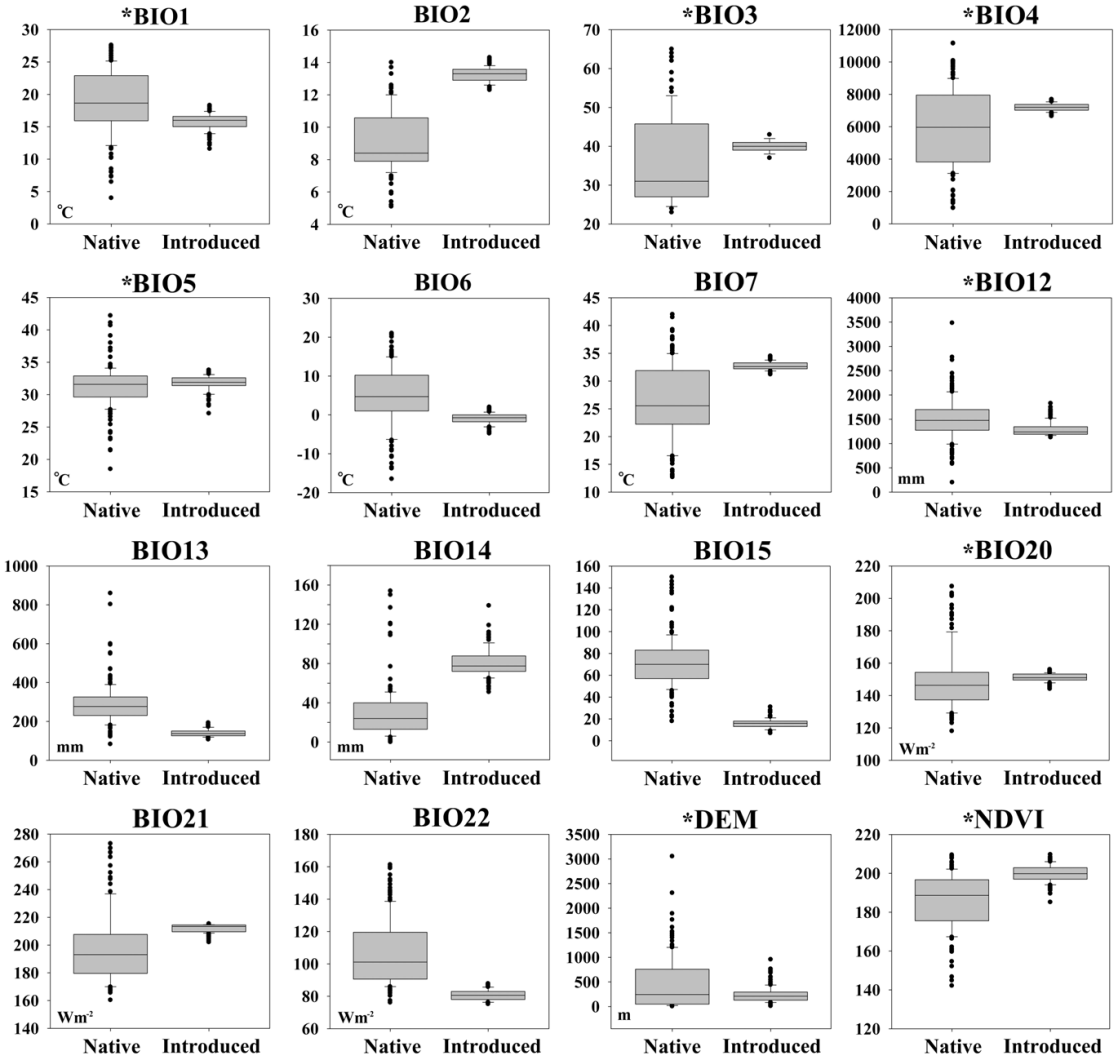
Direct comparison of Bean plataspid occurrence-associated variables between native and introduced distributional areas. The Dataset *I* (variables without asterisk) with columns (i.e., IQR) were not overlapped between the two areas representing variables of high discrepancy. The Dataset *II* (variables with asterisk) with columns were more or less overlapped representing variables of low discrepancy.

Raw environmental data was extracted from environmental rasters at species' occurrence records using ArcGIS 10, and compared in boxplot between native and introduced populations. Boxplot gives a good sense of environmental data distribution (median, minimum, maximum, and the first and third quartiles), that indicate the extent to which the data lies near the median, or near the extremes [44]. Here, the interquartile range (IQR) overlap between native and introduced populations was used to classify the environmental dimensions. The interquartile IQR is a measure of statistical dispersion, it is equal to the difference between the third (Q_3_) and first (Q_1_) quartiles (i.e., IQR = Q_3_−Q_1_), which is represented by column in boxplot. Unlike total range, the IQR is a robust statistic, having a breakdown point of 25%, and is thus often preferred to the total range [44]. All boxplots were prepared in Sigmaplot 11.0 (Systat Software Inc, Chicago, IL, USA), then classified into two datasets by IQRs (or boxplot columns) overlaps between the two populations. Of which, Dataset *I* with IQRs were not overlapped (i.e., BIO 2, 6, 7, 13, 14, 15, 21 and 22), Dataset *II* with IQRs were more or less overlapped (i.e., BIO 1, 3, 4, 5, 12, 20, DEM and NDVI) (Table 1, Figure 1).

Therefore, the Dataset *I* represented environmental dimensions of high discrepancy among the two ranges, and the Dataset *II* represented dimensions of low discrepancy. A principle component analysis (PCA) was then used to visualize the species niche space in reduced dimensions. Two correlation matrixes were prepared for the Dataset *I* and *II* to perform the PCA in SPSS 19 (IBM SPSS Statistics, Chicago, IL, USA). To facilitate visualization, occurrence records were grouped for native and introduced populations respectively. The PCA provided a representation of the bug's niche space across both ranges. The spread of points representing native and invasive populations in the PCA were compared [12], [14]. If the species' niche is retained, invasive occurrences should cluster within the cloud of native range occurrences; separation of the two ranges in PCA space signifies a potential divergence from niche conservation, in a strict sense [22], [45].

### Two variable sets comparison

All models were developed using maximum entropy algorithm implemented in Maxent software (version 3. 3. 3k) [46]–[49]. In exploring areas of potential invasion, another algorithm was used (i.e., GARP, see below). Maximum entropy is a machine-learning technique that predicts species distributions using detailed environmental variables associated with species occurrence. It follows the principle of maximum entropy and spreads out probability as uniformly as possible, but subject to the caveat that they must match empirical information such as known presence. Analysis was run on default program conditions (Logistic output, default convergence threshold (10^−5^) and maximum number of iterations (500)). The logistic output with suitability values ranging from 0 (unsuitable habitat) to 1 (optimal habitat) gives an estimate of probability of presence, assuming that the sampling design is such that typical presence localities have probability of presence of about 0.5 [46], [47]. A jack-knife procedure was used to evaluate the relative importance of each predictor variable and the ability to correctly predict new ranges in the model [50].

Niche models were calibrated against native range environmental data rasters clipped to appropriate size defined by a bounding box containing all known native range occurrences (i.e., the area defined as the geographic space available to the species). Constructed models using the above two environmental datasets were then transferred onto the US (not include Hawaii and Alaska) respectively. Maxent also calculates a Multivariate Environmental Suitability Surface (MESS) map indicating areas where environmental variables occur outside the range of values in the training region, ENM suitability projections in these regions are unreliable, and should be treated cautiously [51]. MESS of native dimensions in contrast to the US (for Dataset *I*) and the world (for Dataset *II*) were prepared.

Area Under Curve (AUC) of the receiver operating characteristic (ROC) plot and omission rate were used for model evaluation. AUC weights the omission error and commission error equally, it is a threshold-independent measure that juxtaposes correct and incorrect predictions over the spectrum of threshold. AUC values range from 0 to 1, where 1 is a perfect fit. Useful models produce AUC values of 0.7–0.9, and models with ‘good discriminating ability’ produce AUC values above 0.9 [52]. Both AUC and omission rate were used for native model evaluation. Success of models transferred to the US to correctly capture Bean plataspid occurrences was tested using omission rate. Omission rate assesses prediction error calculated by the proportion of test points that were not predicted at a particular threshold. We plotted omission rate across the threshold spectrum of Maxent's logistic output values, specifically, we calculated omission rate at the increasing rate of 0.05 degrees against the total 1.0 logistic output.

### Exploring areas of potential invasion

To explore areas of potential invasion globally, the Dataset *II* was used (Table 1). We calibrated models based on native range, and transferred their predictions onto the other continents. Considering that the record in the US does not characterize the actual distribution, and the sample bias in native Asia, we used 89 occurrences of the reduced native sample for model calibration. Although Maxent has appeared superior to GARP in some previous studies [49], carefully assessments of model quality showed no significant differences between the two [53]. Recent studies suggested using multiple algorithms to infer a consensus estimate of niche dimensions [5], [50], [54]–[56]. Hence, we further used the Genetic Algorithm for Rule-set Prediction (GARP, [57]) to explore areas of potential invasion (Text S1). For model evaluation, we calculated binary omission rate of the remaining occurrence (including native and introduced records) at the threshold of the 10th percentile training presence, which assumed a grid cell was suitable if its suitability score was greater than the 10th percentile of training points. The 10th percentile threshold is highly conservative in estimating species' presence and has been more commonly used [2], [58], [59].

## Results

### Direct comparisons

**Figure 2:**
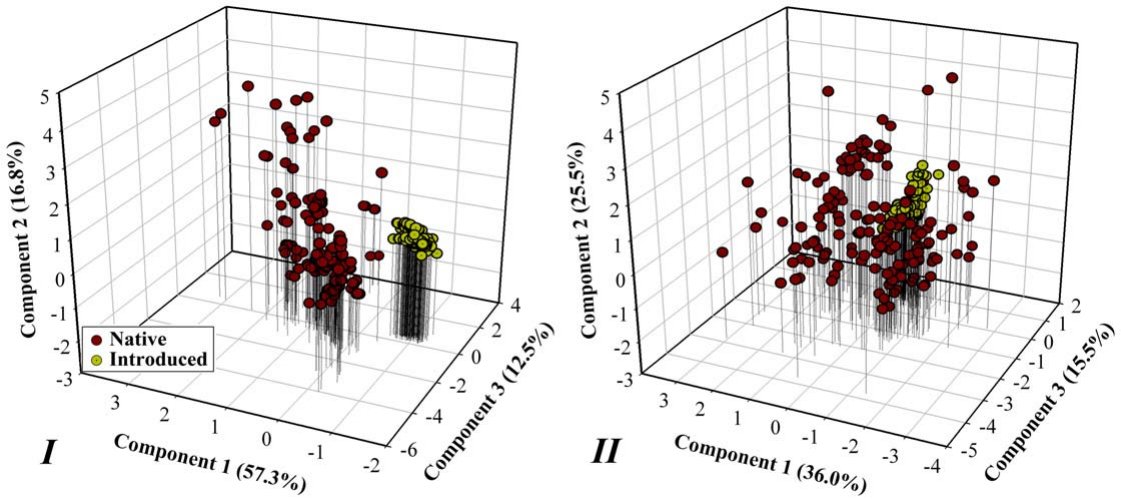
Principal component analysis of Dataset *I* (left) and *II* (right) associated with occurrences of Bean plataspid. Symbols represent Bean plataspid occurrences in native and introduced areas.

Figure 1 summarized the sixteen environmental dimensions and their ranges among native and introduced Bean plataspid populations. PCA of environmental variables associated with Bean plataspid occurrence revealed reduced dimensions that accounted for the observed distribution (Table 1; Figure 2). The first three components were significant and explained 86.59% and 77.04% of the variance in Dataset *I* and *II*, respectively (Table 1). In Dataset *I*, the first component (PC-1) was associated with precipitation seasonality, lowest weekly radiation, and temperature annual range, the second (PC-2) was closely associated with highest weekly radiation, while the third (PC-3) was less clearly associated with single dimension. In Dataset *II*, the first component was associated with annual mean temperature and temperature seasonality, the second was associated with elevation while the third was less clearly associated with single dimension (Table 1). The niche space occupied by the introduced population departed from that occupied by the native population in the reduced dimensions in Dataset *I*, while in Dataset *II*, the orientation of introduced occurrences all fell more closely within those of native population (Figure 2). Comparing these two environmental datasets, one that showed niche difference between native and introduced populations (i.e., Dataset *I*), the other (i.e., Dataset *II*) did not.

### Environmental datasets and model comparisons

**Figure 3:**
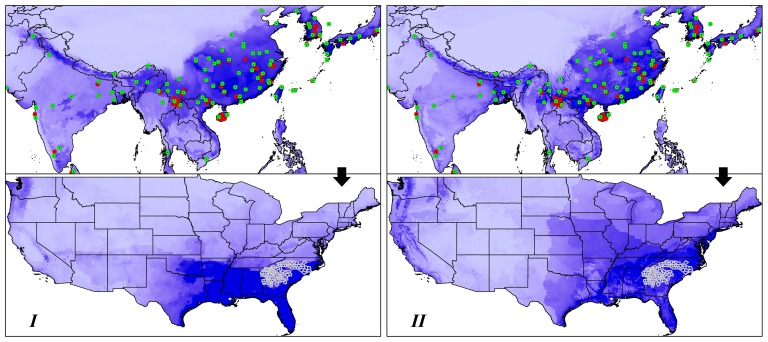
Niche model calibrated on native range and projected onto the US based on Dataset *I* (left) and *II* (right). Dark blue color represents high suitability, light blue indicates low suitability. Green dots indicate the occurrence points used for model calibration, red dots indicate the model testing point, white grids in the US indicate the infested counties.

**Figure 4:**
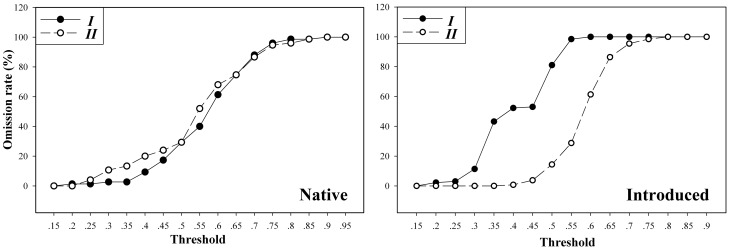
Omission rates among niche models based on Dataset *I* and *II*. Omission rates were plotted in native Asia models and their transferring in the US across the threshold spectrum of Maxent.

In native predictions, models based on the two environmental datasets all showed good model performance compared to random predictions (Dataset *I*: AUC = 0.876; Dataset *II*: AUC = 0.867; Figure 3), and models trained on the two datasets showed little difference in omission rate across Maxent thresholds (Figure 4). However, when transferred onto the US, models based on Dataset *II* showed lower omission rates at the thresholds of 0.2 to 0.75, comparing to that based on Dataset *I*. At the thresholds of 0.25 to 0.5, the omission rates rose significantly in Dataset *I*, but stayed stable at a low level in Dataset *II* (Figure 4), suggesting good model transferability in Dataset *II*. Areas of potential invasion identified by Dataset *I* in the US include extensive areas of the lower Mideast and Southeast, Florida was also identified as suitable in Dataset *I*. In Dataset *II* projected model, the areas identified include most of the infested counties in Georgia, North Carolina and South Carolina, the other states including Mississippi, Alabama, Tennessee and Virginia, and the coastal areas along the Atlantic and Gulf of Mexico (Figure 3). All of these areas with environmental variables were fell in that of the training data (Figure S1, S2).

### Areas of potential invasion

**Figure 5:**
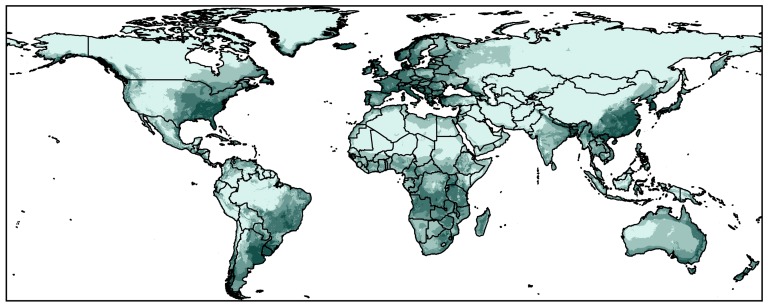
Niche models based on Dataset *II* and transferred worldwide using Maxent. Dark green color represents high suitability, light green indicates low suitability.

Niche models based on the reduced 89 native points yield zero omission (Maxent) and 1.43% omission (GARP) of the independent test points (total 209 points), suggesting good model performance. Comparing to Maxent, the projection of GARP is a little conservative in Europe in contrast to other continents (Figure 5, Figure S3). Outside of native-range areas, high suitable areas identified by both modelling algorithms include the southeastern US in North America, southwestern Europe, southeastern South America, southern Africa, and the eastern coastal Australia (Figure 5, Figure S3). All of these areas with environmental variables were fell in that of the training data (Figure S2). Attention should be paid to quarantine and inspection when engaging in interchanges with the south Asia in these areas.

## Discussion

### Niche difference

Across its native range, the biology of Bean plataspid has been insufficiently studied to adequately understand the ecological constraints of the species [39]–[41], [60]. A species' ecological niche can be characterized through either mechanistic or correlative approaches (i.e., ENM), with the former identifying the physiological determinants of a species' tolerance to environmental conditions, and the later identifying the ecological niche by associating known species' environmental tolerances derived from the conditions found at actual occurrences [7], [18]. Correlative methods are made increasingly possible through the availability of species' occurrence data and geographic information system (GIS) data [6], [7], which are particularly useful for poorly known species, such as Bean plataspid. In PCA, the annual mean temperature, temperature seasonality, temperature annual range, the highest/lowest weekly radiation, and elevation showed their significance in explaining the bug's distribution. In ENMs, the annual precipitation and radiation, temperature seasonality, the maximum temperature of the warmest month, and precipitation of the driest month appeared to be significant in model calibration.

However, care should be taken in interpreting their results, as ENM approaches were based on observations that already include effects of biotic interactions on species distributions [61], and thus recovered the realized or potential niche [20], [62]. This portion of the fundamental niche, limited by biotic interaction or dispersal limitations, is unlikely to capture the full ecological tolerances of the species. In fact, the fundamental niche is rarely fully displayed in geographic space [20], [22], [63]. The spectrum of host plants available to Bean plataspid is limited due to an obligate relationship with a bacterial endosymbiont which allows it to feed on soybean and other legumes [26], [35], [64]. This association might influence the bug's potential for ecological expansion across the invasive range, reducing the occupancy of the range predicted by native range occurrences. It is unlikely that the current invasive range is in a distributional equilibrium, as many areas identified as suitable were not inhabited in the US. Therefore, our observed niche difference can be considered as the realized niche difference, the observed niche space in Dataset *I* and *II* can be considered as the realized niche manifested in two ecological dimensions (i.e., Dataset *I* and *II*).

The ENM here is to characterize the realized niche [20], [62], whereas the PCA is to characterize the realized niche in specific ecological dimensions. In fact, the observed niche difference might tend to happen in the fine resolution environmental dimensions. At small spatial extents, the fundamental niche might express differently because of heterogeneity of the landscape. While at broad spatial scale, the species interactions are weak, diffuse, or non-specific, it will be unusual to see such niche difference [61]. The observed “conserved” or “relaxed” variables [21] might not be simply because of their biological meaningful to the species (i.e., actually restricting species geographical range), but also because whether they were released by the fundamental niche in the heterogeneous landscape in which the species occur. In ecological dimensions, the realized niche difference might be displayed in some environmental axes, but not others [12]. The latter portion dimensions were also biological meaningful and might be more useful for niche model calibration. Actually, the other factors (e.g., sampling bias or environmental data quality) might also play a role in the observed niche difference, although they are not the topic here.

### Environmental dimension and niche model transferability

Environmental variable selection is very important for niche model calibration. Apart from considering the biological factors that may restrict species' distributions, the resolution, extent of study range, and correlation among variables have to be taken into consideration [3], [4]. In particular, comparing the ecological envelopes occupied by native and introduced populations offers useful information for variable selection prior to the modelling [4], [12], [21]. Actually, environmental dimensions had been compared using PCA and metrics summarizing differences between niches [11], [14], [45]. Herein, we found that after direct comparisons in boxplot, the environmental dimensions could be better classified into groups before further comparisons were made in the reduced dimensions of PCA. In the PCA, the realized niche in ecological dimensions of Dataset *II* is less discrepancy than that of Dataset *I* between native and introduced populations. Model projected onto the US based on Dataset *II* showed good model transferability in predicting the invasive occurrences (Figure 4), the niche model transferability here was improved via the PCA selecting environmental variables (i.e., Dataset *II*).

Model transfer outside the native range into the non-analog conditions is a challenge for niche modelling algorithms [65]–[67]. Many model protocols have been proposed to improve niche model transferability [56], [68], [69]. Putting the realized niche in ecological dimensions, the realized niches between native and introduced populations might be the same in some dimensions but different in other dimensions [12], [21]. Niche space comparison using PCA offers useful information on environmental dimensions of low or high discrepancy among ranges prior to the modelling. Our proposal was to select environmental dimensions with low discrepancy to improve niche model transferability among these distributional areas. When transferring niche models beyond the native area onto a larger area (comparing to model calibrating area) or globally, the environmental dimensions with low discrepancy and reduced dimensionality might be preferable (e.g., [4]). Our emphasis of selecting environmental dimensions of low discrepancy to improve niche model transferability by no means diminishes the importance of physiological relevance (e.g., [18]), or the effect of correlativeness of the variables (e.g., [3]).

### Areas of potential invasion

High suitable areas were identified by Dataset *II* based model in the US, including the infested states (i.e., Georgia, North Carolina and South Carolina) and large portions of the surrounding states (i.e., Alabama, Tennessee, Mississippi, Louisiana, and Virginia). Other regions, such as Florida and Louisiana, were not supported by Dataset *II* but by Dataset *I*. Considering the bug's propensity to fly, land on, and get inside vehicles, inspection and quarantine should be considered for these states when engaging commercial activity with the infested states. Currently, the U.S. Department of Agriculture's (USDA) Animal and Plant Health Inspection Service (APHIS) is conducting surveys for the Bean plataspid to determine the extent of infestation in Georgia, North Carolina and South Carolina [31]. Attention should also be paid to the areas of high-suitability that surrounding these infested states (Figure 3), and to areas predicted around the world (Figure 5, S3). This should be especially true for the developed areas where intensive trade activity and commercial interchange might facilitate new invasions.

While large areas of the US are proposed to be suitable to the Bean plataspid, these areas represent niche space without consideration of potential biotic interactions or the species dispersal ability. Many additional factors may influence the successful establishment of a non-indigenous species into a novel community, including existing species richness, competitors, predators, food availability, human footprint and climate similarity compared with the source ecosystem [21]. Although the area predicted as suitable for a species does not necessarily mean it will establish populations there, it does offer useful information for determining areas of potential invasion and spread.

## Conclusion

Sixteen environmental dimensions occupied by native and introduced Bean plataspid populations were compared and classified into two datasets with different degrees of discrepancy among the two ranges. Niche models based on the dataset showing low discrepancy recovered good model transferability in the introduced areas, with low omission in recovering species occurrences. Recommendations were made for selecting biological meaningful environmental dimensions of low discrepancy among populations to improve niche model transferability among these geographically separated areas. When transferring niche model onto a larger area or globally, environmental dimensions with low discrepancy and reduced dimensionality were proposed. Outside of its native range, areas with invasion potential include the southeastern US in North America, southwestern Europe, southeastern South America, southern Africa, and the eastern coastal Australia. In the US, the states surrounding the current infested states (i.e., Georgia, North Carolina and South Carolina), including Alabama, Tennessee, Mississippi, Virginia and Florida should be monitored carefully as a result.

## Supporting Information

Figure S1 **MESS map for Dataset** ***I*** **in model comparison.** Areas in red indicate one or more environmental variables outside the range present in the training data.
(TIF)

Figure S2 **MESS map for Dataset** ***II*** **when transferred the model worldwide.** Areas in red indicate one or more environmental variables outside the range present in the training data.
(TIF)

Figure S3 **Niche models based on Dataset** ***II*** **and transferred worldwide using GARP.** Dark green color represents high suitability, light green indicates low suitability.
(TIF)

Text S1 **GARP protocol in exploring area of potential invasion.**
(DOCX)
