# Supplementary figures and images for: Selecting Biological Meaningful Environmental Dimensions of Low Discrepancy among Ranges to Predict Potential Distribution of Bean Plataspid Invasion
Source: PLoS One. 2012 Sep 25;7(9):e46247. doi: 10.1371/journal.pone.0046247 (PMC3457996; doi:10.1371/journal.pone.0046247)

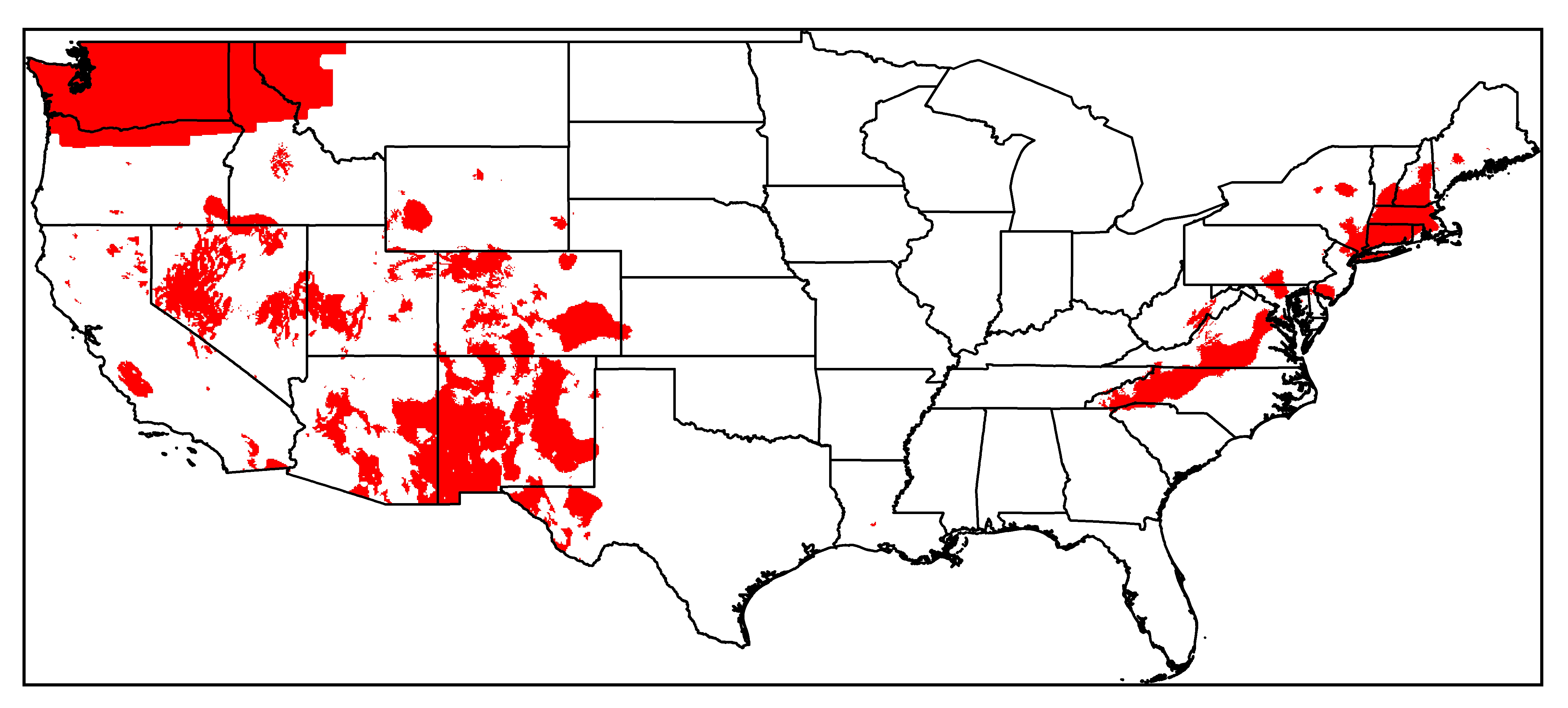

Supplement: Figure S1 — MESS map for Dataset I in model comparison. Areas in red indicate one or more environmental variables outside the range present in the training data. (TIF) [file pone.0046247.s001.tif]

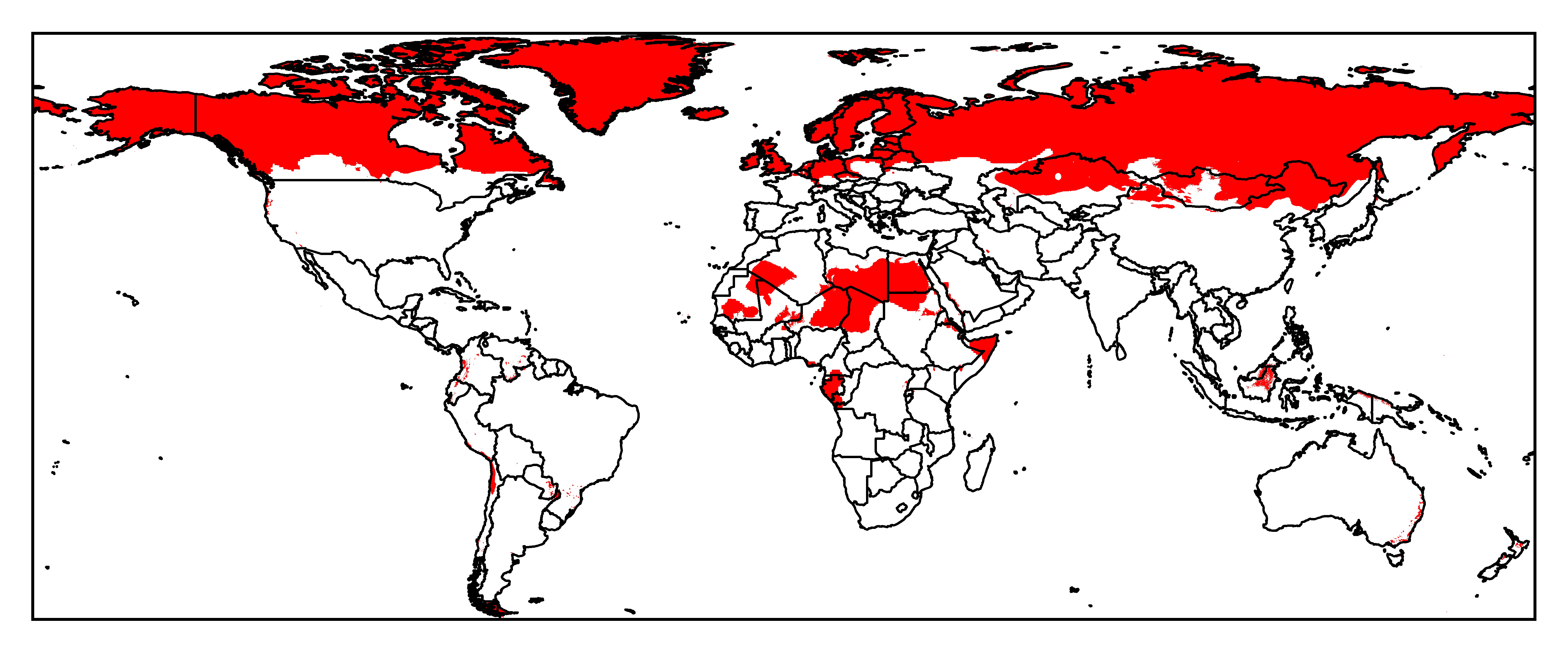

Supplement: Figure S2 — MESS map for Dataset II when transferred the model worldwide. Areas in red indicate one or more environmental variables outside the range present in the training data. (TIF) [file pone.0046247.s002.tif]

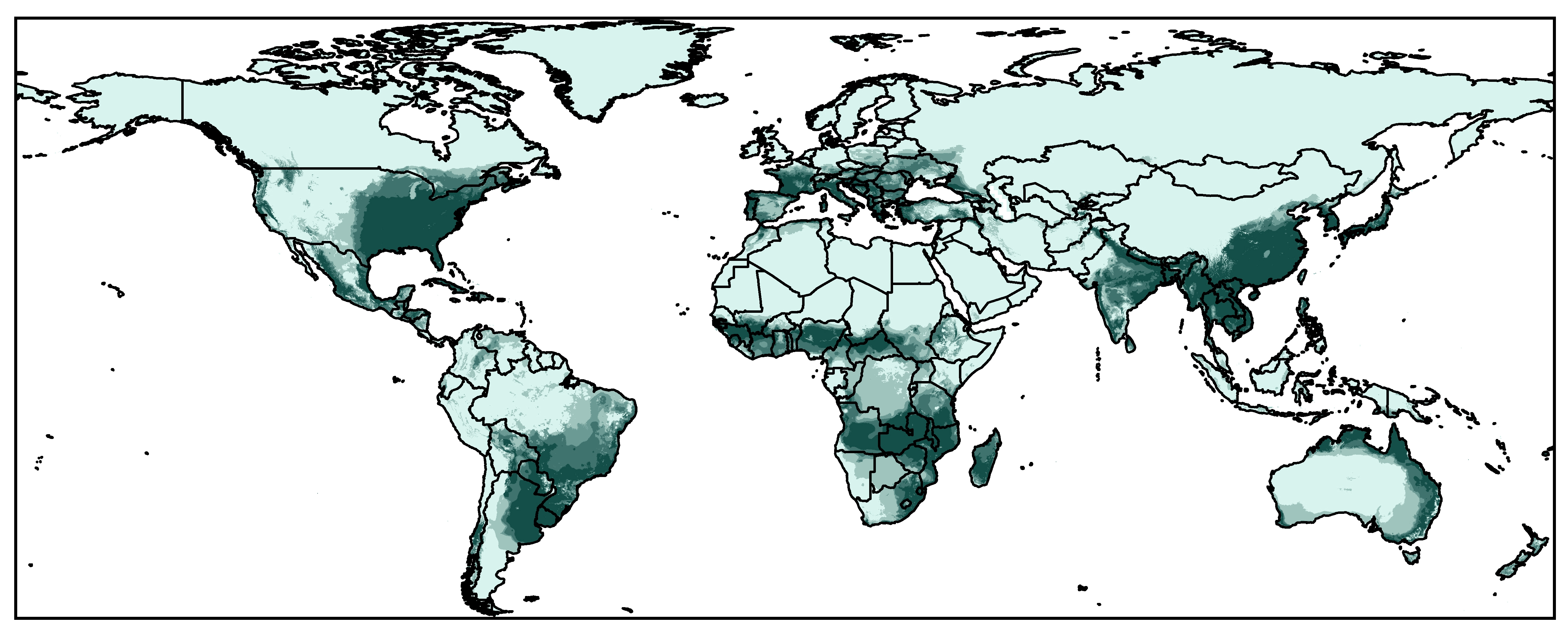

Supplement: Figure S3 — Niche models based on Dataset II and transferred worldwide using GARP. Dark green color represents high suitability, light green indicates low suitability. (TIF) [file pone.0046247.s003.tif]
