# Supplementary material for: Selecting Biological Meaningful Environmental Dimensions of Low Discrepancy among Ranges to Predict Potential Distribution of Bean Plataspid Invasion
Source: PLoS One. 2012 Sep 25;7(9):e46247. doi: 10.1371/journal.pone.0046247 (PMC3457996; doi:10.1371/journal.pone.0046247)
Supplement: Text S1 — GARP protocol in exploring area of potential invasion. (DOCX) [file pone.0046247.s004.docx]

**Text S1 GARP protocol in exploring area of potential invasion.**

GARP uses an evolutionary computing genetic algorithm to search for non-random associations between environmental variables and known occurrences of species, as contrasted with environmental characteristics across the overall study area [1]. Because model development is stochastic, and resulting models can vary in quality, we used a procedure described by Anderson et al. [2] to select an optimal subset of random replicate models for combination as a consensus model. In particular, we developed 100 replicate models based on random subsampling 75% of the 89 input points. Of these models, we retained the 20 with lowest omission error as measured via the remaining 25% of the input points. Then, we retained the 10 models with intermediate commission error. This “best subset” of models were summed to produce final predictions of potential distributions in the form of grids with values ranging from 0 (all models agree in predicting absence) to 10 (all models agree in predicting potential presence).

**References**

1. Stockwell DRB, Peters DP (1999) The GARP modeling system: problems and solutions to automated spatial prediction. International Journal of Geographical Information Systems 13: 143–158.
2. Anderson RP, Lew D, Peterson AT (2003) Evaluating predictive models of species’ distributions: criteria for selecting optimal models. Ecological Modelling 162: 211–232.
